# Impact of follistatin-like 1 on cardiac repair processes following myocardial infarction

**DOI:** 10.3389/fcell.2025.1746363

**Published:** 2026-01-12

**Authors:** Fang He, Xueying Wang, Yongbin Man, Qianqian Xu, Xuejie Yi, Jiao Liu

**Affiliations:** 1 College of Exercise and Health, Shenyang Sport University, Shenyang, China; 2 Department of Kinesiology, Exercise and Health Research Center, Shenyang Sport University, Shenyang, Liaoning, China

**Keywords:** cardiac repair, fibrosis, FSTL1 (follistatin-like 1), gene therapy, inflammation regulation, myocardial infarction

## Abstract

Follistatin-like 1 (FSTL1) is an emerging multifunctional glycoprotein that plays a central role in cardiac repair following myocardial infarction (MI). While previous studies have explored its involvement in modulating inflammation, angiogenesis, and fibrosis, a cohesive mechanistic understanding remains incomplete. In this review, we provide a comprehensive synthesis of current findings and propose an integrated framework in which FSTL1 orchestrates post-infarction healing through multiple signaling cascades, including BMP/SMAD, PI3K/AKT, MAPK, and TGF-β pathways. We highlight its dual actions in both cardiomyocytes and cardiac fibroblasts, as well as its context-dependent interactions with mechanical cues and the immune microenvironment. Recent evidence suggests that FSTL1 may function as a key regulatory hub, coordinating sequential events such as inflammation resolution, extracellular matrix remodeling, and functional recovery. Together, these insights underscore the therapeutic promise of FSTL1 as a molecular target for enhancing cardiac repair and restoring myocardial integrity after infarction.

## Graphical Abstract

**Figure:**
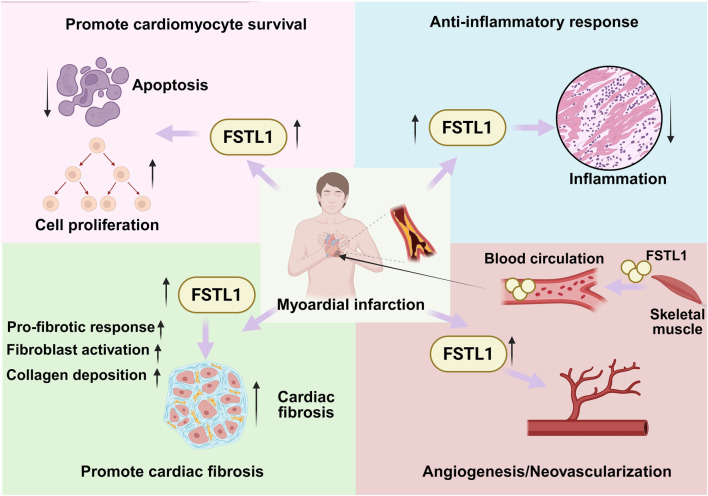

## 1 Introduction

Myocardial infarction (MI) remains one of the leading causes of morbidity and mortality worldwide, despite major advances in reperfusion strategies and pharmacological therapies (Moran et al., 2014; Reed et al., 2017; Martin et al., 2022). Its clinical impact stems not only from the acute loss of cardiomyocytes but also from subsequent maladaptive remodeling that frequently progresses to heart failure (Pfeffer et al., 1991; Tsao et al., 2023; Liu et al., 2024). Cardiac repair after MI is a highly dynamic process involving four overlapping phases: cardiomyocyte death and survival, inflammation, fibrosis, and angiogenesis (Fraccarollo et al., 2012; Viola et al., 2021; Wu et al., 2021). The balance among these events determines whether healing is adaptive or maladaptive. Controlled inflammation and extracellular matrix deposition are essential for preserving structural integrity, whereas excessive fibrosis or persistent inflammation can drive adverse remodeling and progressive dysfunction (Jung et al., 2018; Jiang et al., 2021). Within this context, follistatin-like 1 (FSTL1) has emerged as a key modulator intersecting with multiple stages of repair.

FSTL1 is a secreted glycoprotein originally identified as a TGF-β–inducible gene and is widely expressed, including in the heart. Accumulating evidence indicates that FSTL1 protects cardiomyocytes from apoptosis, shapes inflammatory responses, promotes angiogenesis, and regulates fibroblast activation (Shibanuma et al., 1993; Wei et al., 2015; Xiao et al., 2019; Adams et al., 2007). These pleiotropic actions position it as both a promising therapeutic mediator and a potential biomarker. However, several studies also suggest that FSTL1 can exacerbate pathological fibrosis and adverse remodeling under chronic conditions (Stelzer et al., 2016; Cunningham et al., 2019; Shen et al., 2019), highlighting its context- and time-dependent “double-edged sword” effects.

Despite growing interest, the mechanisms underlying these divergent roles remain poorly defined. Findings from *in vitro* models, animal studies, and clinical observations are not always consistent. More recently, research has expanded to include metabolic disorders and multi-omics profiling (Sundaram et al., 2013; Shi et al., 2016; Parfenova et al., 2021), underscoring broader biological implications that extend beyond the cardiovascular system. In this review, we synthesize current experimental and clinical evidence on FSTL1 in post-MI repair and remodeling, highlight its dual protective and pathogenic roles, and discuss emerging insights from metabolic and systems biology, with the ultimate aim of identifying opportunities for therapeutic translation.

## 2 Basic biology of FSTL1

### 2.1 Gene and protein structure of FSTL1

The human *FSTL1* gene is located on chromosome 3q13.33, is approximately 59 kb in length, and contains 11 exons, the first of which is a noncoding exon (Stelzer et al., 2016; Cunningham et al., 2019). Exons 2–11 encode a 308-amino-acid protein (Parfenova et al., 2021), whereas the 11 th exon also includes the coding sequence for microRNA (miRNA)-198 (miR-198) (Sundaram et al., 2013). Therefore, the primary *FSTL1* transcript produces both the protein and miR-198. Additionally, several miRNA binding sites exist in the 3′UTR region of *FSTL1*, including miR-206, miR-32-5p, and miR-27a. These sites have been shown to functionally inhibit FSTL1 (Shi et al., 2016; Rosenberg et al., 2006; Zhang et al., 2017).

**FIGURE 1:**
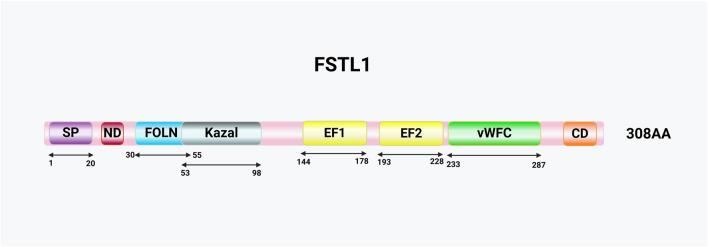
Schematic representation of the FSTL1 protein and its structural domains. Key domains include the signal peptide (SP), N-terminal domain (ND), follistatin-like domain (FOLN), Kazal-like domain (Kazal), EF-hand domains (EF1/2), von Willebrand factor type C domain (vWFC), and the C-terminal domain (CD) enriched with acidic L-amino acids.

FSTL1 is classified within the SPARC family of proteins because of its structural domain similarity to secreted acidic cysteine-rich proteins (SPARC/BM-40/osteoconjugate proteins), which feature the two hallmark structural domains (Chaly et al., 2014a). The first domain is a follistatin-like domain that closely resembles that of follistatin (Figure 1) (Li et al., 2013). Follistatin inhibits the biosynthesis and secretion of follicle-stimulating hormones by binding to activin (Phillips and de Kretser, 1998). Activin, as a member of the TGFβ protein superfamily, not only plays a pivotal role in reproductive biology but also functions as a multifunctional protein, regulating diverse physiological processes, including inflammation (Phillips et al., 2005; Chang et al., 2002; Bamberger et al., 2005; Mayer et al., 2012). Activin levels are elevated in many clinical inflammatory diseases (Mayer et al., 2012) and play a key regulatory role in immune responses. This illustrates the potential role of the follistatin-like domain in regulating activin activity as well as the importance of FSTL1 in regulating immune responses. However, there are conflicting reports on the ability of FSTL1 to bind to activin. Some studies have indicated that FSTL1, unlike follistatin, does not bind to activin (Kawabata et al., 2004). FSTL1 contains a single follistatin-like domain, whereas follistatin contains three such domains (Chaly et al., 2014a). However, other studies have shown that FSTL1 can bind to activin (Zhou et al., 2006), and Tanaka et al. further confirmed using Biacore technology that FSTL1 binds to activin with low affinity and inhibits the interaction between activin and its receptor ActR-IIB (Tanaka et al., 2010). Despite these findings, the binding of FSTL1 to activin requires further *in vivo* validation.

Another key structural domain in FSTL1 is the extracellular calcium-binding domain (Figure 1). In the SPARC family, calcium binding is typically mediated by a pair of EF-hands; however, in FSTL1, this domain is non-functional (Hambrock et al., 2004). This suggests that FSTL1 has unique properties, despite its sequence similarity to other SPARC family members. In addition, FSTL1 contains a vascular hemophilic factor C-type domain, which is absent in other SPARC proteins, and a Kazal-like domain (Figure 1) (Du et al., 2024). Serine protease inhibitors, which often contain Kazal domains, are widespread in animals and are crucial for maintaining normal physiological and cellular functions (Rimphanitchayakit and Tassanakajon, 2010). The complex structure and multi-specific binding capabilities of FSTL1 suggest that it plays a broad range of physiological and pathological roles.

### 2.2 Role of FSTL1 in normal cardiac physiology

The role of FSTL1 in cardiac physiology is not limited to cardiomyocytes but extends to the broader cardiovascular system. FSTL1 expression has been observed in various cell types, including fibroblasts, epicardial mesothelial cells, skeletal and smooth muscle cells, and vascular endothelial cells in myocardial vessels (Wei et al., 2015; Miyamae et al., 2006; Wilson et al., 2010; Ouchi et al., 2008; Lara-Pezzi et al., 2008). FSTL1 has been identified as a cardiac factor (Oshima et al., 2008), playing a regulatory role in cardiac development and cardiomyocyte proliferation and differentiation. FSTL1 is widely expressed during early development in mice and chicks. However, during mid-gestation, its expression is localized to non-myocardial components and continues to be present in adulthood (Wei et al., 2015; Adams et al., 2007; van den Berg et al., 2007). However, recent studies have found that FSTL1 is primarily expressed in cardiac fibroblasts in the hearts of adult mammals, with no detectable presence of FSTL1 in the epicardium (Kretzschmar et al., 2018). These findings suggest that FSTL1 plays a regulatory role in the initial and late stages of cardiomyocyte formation (Horak et al., 2022). WNT signaling is essential for normal heart development and requires precise temporal and spatial regulation (Stylianidis et al., 2017). Notably, FSTL1 regulates the WNT pathway (Sundaram et al., 2017). In experiments where there was a specific deletion of *FSTL1* in cardiomyocytes or cardiac fibroblasts or overexpression of FSTL1 in the heart and skeletal muscle of mice, no baseline phenotypic or cardiac functional changes were observed under normal physiological conditions. However, FSTL1 exhibited significant effects after cardiac injury in both scenarios (Kretzschmar et al., 2018; Shimano et al., 2011; Tanaka et al., 2016; Maruyama et al., 2016). Additionally, FSTL1 is involved in the vascularization of the cardiovascular system. Through exercise-induced secretion from skeletal muscle, FSTL1 promotes cardiac angiogenesis (Xi et al., 2021). During the development of cardiovascular diseases, the expression of FSTL1 is closely related to cardiovascular pathology; therefore, an in-depth understanding of its biological functions is important for the treatment and prevention of cardiovascular diseases.

### 2.3 Factors influencing the expression of FSTL1

FSTL1 expression is regulated by a variety of physiological and systemic factors, including age, sex, adiposity, and immune interactions, which can influence its cardioprotective effects, particularly after myocardial infarction (MI). While studies on FSTL1 in cardiovascular diseases are limited, other pathological conditions have provided useful insights. For instance, both age and sex can influence FSTL1 levels, with hormonal and age-related changes affecting its expression and function (Wang et al., 2011; Zhong et al., 2022). Additionally, adiposity and obesity-related inflammation modulate FSTL1 expression, with obesity generally leading to altered secretion patterns, although inflammation can upregulate its levels (Miyamae et al., 2006). FSTL1 also plays a key role in regulating the inflammatory microenvironment, which is crucial for effective tissue repair. Notably, immune interactions, such as macrophage recruitment and neuroimmune signaling, also regulate FSTL1 expression, suggesting that FSTL1 may play a role in heart repair by modulating immune responses and neural signaling (Liu et al., 2025; Zheng et al., 2025). These regulatory effects could influence the efficiency of cardiac recovery and provide new insights for developing more effective heart repair therapies.

### 2.4 Epigenetic regulation of FSTL1 in cardiac repair

Recently, the role of epigenetic regulation in cardiac pathophysiology has attracted widespread attention. For FSTL1, changes in the expression of specific miRNAs and alterations in DNA methylation have been shown to significantly affect expression levels and functions during myocardial repair. Epigenetic mechanisms play a crucial role in regulating the structural and functional remodeling of the heart during the repair process following MI. Studies have shown that FSTL1 in the epicardium induces epithelial-mesenchymal transition (EMT), stemness, and epicardial-mesothelial cell migration through an miR-200c-3p–dependent pathway (Pontemezzo et al., 2021). EMT is a key process in cardiac development and repair, particularly after MI, where it contributes to the remodeling and repair of damaged myocardial tissue. miR-200c-3p plays a crucial role in the regulation of this process. Specifically, during EMT in epicardial cells, the expression of miR-200c-3p is significantly downregulated, leading to a marked upregulation of its target, FSTL1. Conversely, overexpression of miR-200c-3p can inhibit TGF-β1-mediated upregulation of FSTL1, thereby suppressing EMT and its migratory characteristics. This regulatory mechanism offers a potential therapeutic strategy for cardiac repair and regeneration, suggesting that targeting miRNA regulation may be an important strategy for post-MI treatment.

In addition to miR-200c-3p, another miRNA that inhibits FSTL1 expression in the heart is miR-9-5p. Research has shown that this miRNA modulate FSTL1 expression, thereby suppressing cell death and oxidative stress, ultimately mitigating the cardiac remodeling process following MI (Xiao et al., 2019). This mechanism highlights the potential of miRNA regulation to reduce myocardial injury and aid in the recovery of cardiac function. Furthermore, DNA methylation, a critical epigenetic modification, may play a significant role in regulating FSTL1 expression. In this regard, methylation of the *FSTL1* promoter may dynamically influence its expression, although the spatiotemporal pattern and functional impact of this mechanism during post-MI repair remain to be established. Notably, miRNA-198 and FSTL1 are derived from the same primary transcript yet exhibit mutually exclusive expression patterns under pathological stimuli (Sundaram et al., 2013). This reciprocal relationship suggests a finely tuned regulatory switch, whereby the balance between miRNA-198 and FSTL1 may determine the cellular response to stress or injury.

In summary, through epigenetic regulation, particularly by targeting miRNAs or modifying DNA methylation status, precise control of *FSTL1* expression can be achieved. This not only enhances myocardial repair following infarction but also offers new avenues and potential applications for cardiac disease treatment. Future studies should explore the relationship between these epigenetic mechanisms and MI repair to better apply these regulatory strategies, thereby providing more robust support for personalized therapeutic approaches.

## 3 Role of FSTL1 after myocardial infarction

Myocardial infarction leads to massive cardiomyocyte loss, inflammation, and structural remodeling, ultimately impairing cardiac function. Growing experimental evidence indicates that FSTL1 is centrally involved in orchestrating these processes. To place this evidence into perspective, Table 1 compiles findings from *in vitro*, animal, and human studies that have examined the role of FSTL1 in post-MI repair. By bringing together diverse models, this overview provides a foundation for understanding the multifaceted actions of FSTL1 before turning to detailed mechanistic pathways.

**TABLE 1:** Key evidence on FSTL1 in post-MI cardiac repair and remodeling (preclinical and clinical).

Model/sample	Intervention/context	Main findings	References
Preclinical MI/I-R models			
*In vitro* (cardiomyocytes/fibroblasts)	Recombinant FSTL1 protein; knockdown/overexpression	Activates AMPK–ACC; limits apoptosis; modulates fibroblast activation and ECM synthesis	Oshima et al. (2008), Maruyama et al. (2016)
*In vitro* (endothelial cells)	Recombinant FSTL1 protein	Promotes endothelial proliferation/migration/tube formation (see Section 3.5 ; Figure 3)	Ouchi et al. (2008)
Mouse MI model (cfKO)	Cardiomyocyte-specific deletion of FSTL1	Increased cardiac rupture; impaired fibroblast activation and scar formation	Maruyama et al. (2016)
Mouse MI model (therapy)	Epicardial patch delivering rFSTL1; MSC therapy enhanced with FSTL1	rFSTL1 patch improved cardiac function and angiogenesis; FSTL1-enhanced MSCs augmented repair efficacy	Wei et al. (2015), Shen et al. (2019)
Rat MI model (exercise intervention)	Dynamic resistance exercise	Exercise-induced FSTL1 promoted angiogenesis via DIP2A-dependent signaling (see Section 3.5/Figure 3)	Xi et al. (2021)
Clinical MI/HF observations			
Human (MI/HF biomarkers)	Circulating FSTL1 levels in patients with MI or HF	Elevated FSTL1 associated with adverse LV remodeling and increased risk of major adverse cardiovascular events; correlations with clinical risk markers reported	Aikawa et al. (2019), Uematsu et al. (2020)
Mechanistic receptor evidence			
Mechanistic receptor evidence	FSTL1 binding studies	DIP2A identified as functional receptor mediating cardioprotective and angiogenic effects	Tanaka et al. (2010)
Systems-level evidence in post-MI remodeling			
Systems biology/multi-omics	Multi-omics profiling in MI and cardiac remodeling	Metabolic stress and inflammatory remodeling networks in post-MI settings	Liu et al. (2024)

Abbreviations: ECM, extracellular matrix; LV, left ventricle; MI, myocardial infarction; HF, heart failure.

As summarized in Table 1, evidence across experimental models supports that FSTL1 participates in multiple facets of post-MI repair, including suppression of apoptosis and inflammatory injury, promotion of angiogenesis, and modulation of fibroblast activation and extracellular matrix remodeling. Preclinical studies highlight its potential as a therapeutic mediator, while clinical observations suggest that circulating FSTL1 is associated with adverse left ventricular remodeling in MI/HF settings. Recent multi-omics analyses in post-MI remodeling further extend these findings by placing FSTL1 within broader regulatory networks that integrate inflammatory and remodeling programs at a systems level. At the same time, discrepancies remain: FSTL1 often appears protective during the acute repair window but may contribute to fibrotic remodeling when signaling is sustained. In the sections that follow, we discuss these mechanisms in greater detail within a stage-based framework.

### 3.1 Cell type–specific mechanisms of FSTL1 in major cardiac cell populations

A While FSTL1 is frequently discussed as a single cardiokine, accumulating evidence indicates that its downstream effects are strongly cell type–dependent in the infarcted heart. Cardiomyocytes, endothelial cells, cardiac fibroblasts, and infiltrating immune cells form a dynamic network in which FSTL1 acts as both a paracrine effector and a microenvironmental modulator. A cell type–centered summary therefore helps reconcile divergent observations across models and clarifies why FSTL1 effects are often time dependent after MI.

**FIGURE 2:**
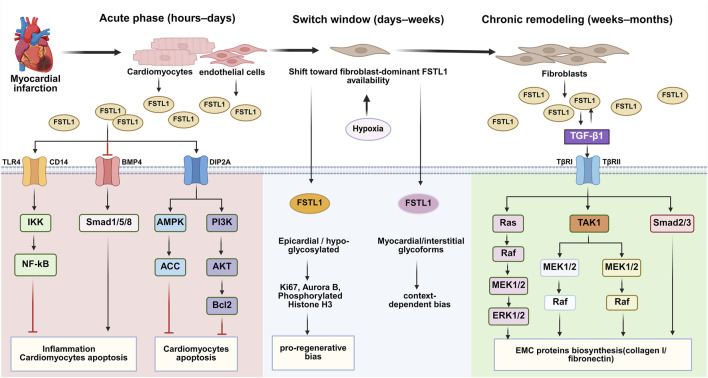
Time-linked switch model for the dual role of FSTL1 after myocardial infarction. Early FSTL1 (cardiomyocyte/endothelial-dominant) promotes cardioprotection and angiogenic repair (e.g., AMPK/PI3K–Akt; DIP2A-associated signaling), whereas late FSTL1 (fibroblast-dominant) biases toward profibrotic remodeling (e.g., TGF-β-related pathways and MAPK cascades). Microenvironmental cues and FSTL1 glycosylation state modulate receptor/pathway selection across phases.

Cardiac fibroblasts (scar formation and remodeling). Fibroblasts constitute a major source of FSTL1 in the adult injured heart and represent a key responder population during repair. Experimental evidence shows that FSTL1 supports early “reparative” fibroblast activation by enhancing fibroblast migration and proliferation, a process linked to scar stabilization and reduced risk of post-MI rupture. Mechanistically, these pro-repair fibroblast responses have been attributed primarily to ERK1/2 signaling (see Section Proliferation/Fibroblast Activation Stage and Figure 2) (Maruyama et al., 2016). In parallel, other studies suggest that FSTL1 may amplify profibrotic programs by interacting with the TGF-β axis and downstream Smad-dependent and MAPK pathways, thereby promoting myofibroblast differentiation and extracellular matrix accumulation. Together, these findings support a context- and phase-dependent interpretation: short-term fibroblast-directed FSTL1 signaling may be required for structurally competent healing, whereas sustained or excessive signaling could contribute to maladaptive fibrosis during chronic remodeling.

**FIGURE 3:**
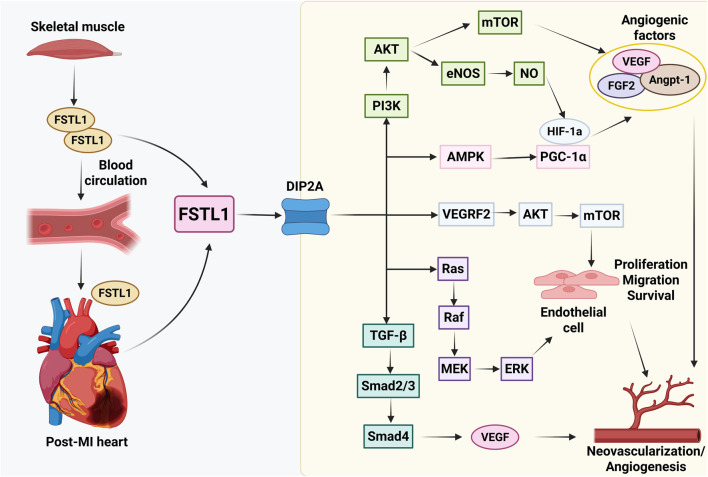
Pro-angiogenic mechanisms of FSTL1 in post-MI cardiac repair. FSTL1 derived from the heart and skeletal muscle enters the circulation and binds to its receptor DIP2A on endothelial cells. Downstream, the DIP2A–PI3K/Akt/eNOS–NO axis and the AMPK–HIF-1α/PGC-1α module have been implicated in promoting endothelial cell proliferation, migration, survival, and angiogenic factor expression (VEGF, FGF2, Angpt-1), ultimately enhancing neovascularization. Additional proposed branches (e.g., VEGFR2-associated signaling, Ras/MEK/ERK, and TGF-β/Smad2/3) are indicated as reported mechanisms. Black arrows represent activation or upregulation.

Endothelial cells (angiogenesis and vascular protection). In endothelial cells, FSTL1 consistently exhibits pro-survival and pro-angiogenic actions. *In vitro*, FSTL1 enhances endothelial migration, network formation, and resistance to apoptotic stress, and *in vivo* it accelerates revascularization in ischemic settings. Mechanistically, these endothelial outputs are largely mediated by DIP2A-dependent pro-survival/pro-angiogenic signaling (see Pro-angiogenic role of FSTL1 and Figure 3). Receptor-level evidence further indicates that DIP2A can function as a FSTL1-binding partner that mediates key endothelial outputs, including Akt activation and angiogenic behavior (Ouchi et al., 2010). These endothelial mechanisms provide a cellular basis for the improved perfusion and neovascularization observed in multiple post-MI interventions involving recombinant FSTL1, gene delivery, or exercise-associated FSTL1 induction.

Macrophages and innate immune cells (inflammation shaping and resolution). The post-MI inflammatory response is dominated by monocytes/macrophages, and FSTL1 has been reported to modulate macrophage inflammatory programs. In ischemia/reperfusion and cultured macrophage settings, FSTL1 can suppress pro-inflammatory gene expression through AMPK-dependent mechanisms, suggesting a potential role in limiting inflammatory injury and supporting the transition toward repair (Ogura et al., 2012). Conversely, other immune studies report that FSTL1 can promote inflammatory outputs, including enhancement of NLRP3 inflammasome-related signaling and IL-1β production, implying that macrophage responses to FSTL1 may be highly dependent on stimulus, tissue context, and timing (Chaly et al., 2014b). In the infarcted heart, this dual potential supports the broader “time-linked switch” model: early FSTL1 activity may favor controlled inflammation and repair, whereas persistent immune activation could contribute to chronic remodeling.

Taken together, these cell type–specific mechanisms support a unified view of FSTL1 biology after MI: endothelial and cardiomyocyte-associated cytoprotective/angiogenic programs are most beneficial during the acute–subacute repair window, while fibroblast-dominant programs may increasingly shape late remodeling and fibrosis risk. This framework also underscores why translational strategies targeting FSTL1 must explicitly account for cell-specific delivery, dosing, and timing.

### 3.2 Myocardial ischemia and early damage control

In the early injury phase following myocardial infarction (from a few hours to days), the sudden interruption of coronary blood flow leads to a rapid collapse in the energy metabolism of cardiac cells. Oxidative phosphorylation is blocked, ATP production decreases significantly, and lactate accumulates. This results in energy depletion and disruption of the cell membrane’s homeostasis, ultimately causing dysfunction and necrosis of cardiac cells (Braasch et al., 1968; Kübler and Spieckermann, 1970; Frangogiannis, 2015). This is followed by an intense inflammatory response, which is the body’s initial reaction to clear dead cells and matrix debris.

At the peak of the inflammatory response, cardiac cells activate survival signals to resist ischemic stress. The IKK/NF-κB signaling pathway protects cardiac cells through multiple mechanisms (Figure 2). Under hypoxic conditions, NF-κB inhibits mitochondrial damage by activating the IKK pathway, preventing the opening of the mitochondrial permeability transition pore (PTP), loss of membrane potential, and release of pro-apoptotic factors, thereby reducing cardiac cell death (Regula et al., 2004). Additionally, under pressure overload conditions, NF-κB reduces oxidative stress by regulating the expression of antioxidant enzymes like manganese superoxide dismutase (MnSOD) and inhibits the activation of pro-apoptotic factors like JNK, further reducing apoptosis and cardiac remodeling (Hikoso et al., 2009). Meanwhile, the BMP4/Smad1/5/8 signaling axis is also regulated in the early stages (Figure 2). BMP4, a member of the TGF-β superfamily, promotes pro-inflammatory and cell death responses by activating the downstream Smad1/5/8 signaling pathway. After myocardial infarction, BMP4 signaling is upregulated in the infarct border zone, potentially promoting local inflammation and apoptosis. However, FSTL1, as an antagonist of BMP4, can inhibit BMP4-induced phosphorylation of Smad1/5/8, thus alleviating inflammation and protecting cardiac cells from further damage. This mechanism is crucial for myocardial protection (Ogura et al., 2012). In addition, FSTL1 inhibits apoptosis in cardiac and endothelial cells by activating AMPK-dependent and Akt-associated pro-survival signaling (Ogura et al., 2012; Liu et al., 2010). Through the AMPK pathway, FSTL1 reduces cell damage caused by hypoxia/reoxygenation. Meanwhile, through the PI3K/Akt pathway, FSTL1 upregulates Bcl2 proteins and reduces apoptosis in endothelial cells induced by oxidized low-density lipoprotein (ox-LDL), thereby exerting a cell-protective effect. Therefore, in this early phase, the inflammatory response and anti-apoptotic signals are intertwined, attempting to clear necrotic tissue while simultaneously activating anti-apoptotic and protective mechanisms to limit the expansion of damage. This sets the stage for the subsequent proliferative/repair phase.

### 3.3 Proliferation/fibroblast activation stage

In the proliferative and repair phase following myocardial infarction, the activation of fibroblasts in the cardiac repair process is critical for myocardial remodeling. FSTL1 plays a key role in this phase, particularly in promoting the proliferation, migration, and fibrosis of cardiac fibroblasts. Studies have shown that FSTL1 promotes the proliferation and migration of fibroblasts by activating the ERK1/2 signaling pathway, which supports the synthesis of extracellular matrix (ECM) that is crucial for scar formation in the infarcted region (Maruyama et al., 2016). TGF-β is an important pro-fibrotic factor, widely involved in regulating fibroblast proliferation, transformation, migration, and ECM production. The TGF-β/Smad signaling pathway is the classic signaling pathway in myocardial fibrosis (Sibinska et al., 2017). After binding with TGF-β1 or its type II receptor (TβRII), FSTL1 enhances the expression of α-smooth muscle actin and the synthesis of type I collagen and fibronectin by regulating the phosphorylation of downstream Smad2/3, while inhibiting collagen degradation through the TGF-β/Smad signaling pathway (Figure 2). In addition to the classic Smad pathway, FSTL1 also functions through non-classical Smad pathways, with the mitogen-activated protein kinase (MAPK) signaling pathway being the most important. Specifically, FSTL1 can upregulate the MAPK signaling pathway through TGF-β1, activating p38, JNK, and ERK signals, thereby promoting fibroblast proliferation and differentiation, and inducing tissue fibrosis (Figure 2) (Maruyama et al., 2016; Jin et al., 2018). Furthermore, FSTL1 is a secreted glycoprotein, and its biological activity depends on its glycosylation status. The low-glycosylated form of FSTL1 promotes myocardial cell proliferation by activating markers of myocardial cell proliferation (such as Ki67, Aurora B, phosphorylated histone H3, etc.), whereas the high-glycosylated form of FSTL1 does not show the same proliferative effect (Figure 2) (Magadum et al., 2018; Kerkelä, 2022). Under hypoxic conditions (such as after myocardial infarction), the secretion of FSTL1 by human cardiac fibroblasts increases, and the low-glycosylated form of FSTL1 promotes better regenerative effects, particularly in enhancing cell proliferation (Peters et al., 2022). This finding suggests that using FSTL1 with different glycosylation forms could significantly improve the therapeutic effects of cardiac regeneration.

In summary, FSTL1 is essential for cardiac fibroblasts to synthesize ECM components, especially during scar formation and repair after MI, contributing to the maintenance of cardiac structural stability and functional recovery.

### 3.4 The dual role of FSTL1 in post-myocardial infarction: a time-linked switch model

FSTL1 is a stress-induced secreted protein that is markedly upregulated after MI. Accumulating evidence supports a unified interpretation of its apparent “duality”: FSTL1 is predominantly cardioprotective during the acute–subacute repair window but can become pro-fibrotic during chronic remodeling. We propose that this behavior reflects a time-linked switch driven by shifts in dominant cellular sources, changes in post-translational modification (notably glycosylation), evolving receptor availability/usage, and microenvironmental cues (inflammation, hypoxia, mechanical load, and metabolic state) that collectively re-route downstream signaling outputs.

Acute–subacute phase (hours to days): cardiomyocyte/endothelial-dominant protective signaling. In the early phase after MI, cardiomyocytes and endothelial cells contribute substantially to FSTL1 production. In this context, FSTL1 preferentially engages pro-survival and stress-adaptation programs (e.g., AMPK and PI3K/Akt-associated signaling), limiting apoptosis and dampening excessive inflammatory amplification. In endothelial cells, FSTL1–DIP2A signaling supports vascular repair by promoting endothelial survival, migration, and angiogenic response (Figure 3) (Karisa et al., 2025). Consistent with a “repair-window” role, physiological interventions such as exercise can increase circulating FSTL1 and are associated with enhanced post-MI reparative signaling and improved functional recovery, although the dominant downstream axes and their net impact on fibrosis likely depend on timing and disease context.

Transition to chronic remodeling (weeks): fibroblast-dominant profibrotic bias. As debris clearance proceeds and the infarct matures, fibroblasts and myofibroblasts expand and become major producers (and targets) of FSTL1 within the infarct/border zones under persistent hypoxia and inflammatory signaling. During early scar formation, FSTL1 can support reparative fibrosis by promoting fibroblast proliferation and migration through ERK1/2 signaling, facilitating timely extracellular matrix (ECM) deposition and structural stabilization (Maruyama et al., 2016). However, with sustained activation in the chronic phase, fibroblast-centered signaling increasingly favors myofibroblast differentiation and ECM accumulation, shifting the net effect toward maladaptive fibrosis and adverse remodeling.

Determinants of the switch: glycosylation, receptor landscape, and microenvironmental cues. A key molecular determinant is glycosylation status. Low-glycosylated FSTL1 (e.g., epicardial-associated forms) has been linked to regenerative/proliferative signaling, whereas more highly glycosylated myocardial-derived forms are more consistently associated with cytoprotection and ECM-related outputs; stress-driven changes in glycosylation patterns may therefore bias FSTL1 activity toward pro-fibrotic remodeling (Xi et al., 2016). In parallel, the receptor network is likely dynamic: DIP2A represents a validated mediator of several cardiovascular protective/angiogenic outputs, while additional binding partners/receptors (e.g., TLR4-related innate immune signaling or other proposed interactors) have been suggested but remain insufficiently validated in specific cardiac cell types and disease stages (Karisa et al., 2025). Finally, mechanical overload, persistent inflammation, and metabolic disturbances (e.g., diabetes) can reshape the myocardial milieu and may reprogram FSTL1 downstream signaling, thereby accelerating the transition from protective repair to pathological remodeling (Lu et al., 2021).

This time-linked switch model provides a mechanistic framework that reconciles divergent findings across models and generates testable predictions for translation: the therapeutic benefit–risk profile of FSTL1 should depend on timing, cell-targeted delivery, and glycoform control (Figure 2).

### 3.5 Pro-angiogenic role of FSTL1

Angiogenesis is essential for cardiac recovery following MI during the heart tissue repair phase. In addition to its role in modulating cardiomyocyte survival, inflammation, and fibrosis, FSTL1 plays a pivotal role in promoting angiogenesis during post-MI cardiac repair. FSTL1 is a cardiokine secreted not only by the heart but also by other organs, such as skeletal muscles and adipose tissues (Miyabe et al., 2014; Xu et al., 2020). Recent studies have demonstrated that skeletal muscle-derived FSTL1 can reach the heart via the circulation and exert pro-angiogenic effects (Xi et al., 2021; Xi et al., 2019). Therefore, cardiac- and muscle-derived FSTL1 synergistically contribute to the enhancement of cardiac angiogenesis and myocardial repair.

Mechanistically, FSTL1 exerts its proangiogenic effects primarily by binding to its receptor DIP2A in endothelial and smooth muscle cells (Xi et al., 2021; Xi et al., 2019). Downstream, a DIP2A–PI3K/Akt/eNOS–NO axis and an AMPK–HIF-1α/PGC-1α module have been implicated in endothelial proliferation, migration, survival, and angiogenic factor induction (see Figure 3). Additional proposed branches (e.g., TGF-β/Smad2/3- and ERK-related signaling) are summarized in Figure 3 and are not discussed in detail here.

Mechanistically, FSTL1 exerts its proangiogenic effects primarily by binding to its receptor DIP2A in endothelial and smooth muscle cells (Xi et al., 2021; Xi et al., 2019). Among the downstream programs, the PI3K/Akt/mTOR pathway enhances the expression of VEGF, FGF2, and angiopoietin-1, thereby facilitating vessel formation and stabilization (Figure 3) (Wilson et al., 2010; Arabzadeh et al., 2020). PI3K/Akt also phosphorylates eNOS, leading to NO production, which in turn induces HIF-1α and promotes the expression of VEGF, FGF2, and angiopoietin-1 (Figure 3) (Xi et al., 2021; Ouchi et al., 2010). Additionally, AMPK activation, which is regulated under injury conditions such as ischemia, infarction, and exercise, induces HIF-1α and PGC-1α, thereby directly promoting the expression of various angiogenic growth factors (Figure 3) (Shimano et al., 2011; Xi et al., 2016; Hayakawa et al., 2015). Additional proposed branches (e.g., TGF-β/Smad2/3- and ERK-related signaling) are summarized in Figure 3 and are not discussed in detail here.

Collectively, these multilevel signaling mechanisms enable FSTL1 to function as an upstream regulator of angiogenesis, orchestrate endothelial responses, and enhance neovascularization within ischemic myocardium. Preclinical studies using recombinant FSTL1 delivery, FSTL1-overexpressing models, and exercise interventions have consistently demonstrated improved cardiac angiogenesis, reduced fibrosis, and enhanced functional recovery post-MI. In contrast to traditional angiogenic factors, such as VEGF, FSTL1 exhibits unique pro-angiogenic mechanisms and anti-inflammatory properties that may synergistically enhance vascular repair and stabilization after MI (Shimano et al., 2011; Hu et al., 2020).

### 3.6 Chronic remodeling phase

Cardiac fibroblasts undergo significant phenotypic changes after myocardial infarction, becoming the dominant cell type in the healing infarct region. As dead cells are cleared, fibroblasts transform into a synthetic myofibroblast phenotype, significantly increasing the synthesis of extracellular matrix (ECM) proteins (Shinde et al., 2014). During this process, myofibroblasts synthesize and secrete large amounts of collagen, fibronectin, and other matrix proteins (Shinde et al., 2014; Squires et al., 2005). These proteins are core components of scar tissue, helping to repair the damaged myocardium and restore the mechanical stability of the heart. However, excessive myocardial fibrosis and ECM deposition increase the stiffness of the ventricular wall, leading to diastolic dysfunction and ultimately a decline in heart function, which can even progress to heart failure (Davis and Myofibroblasts, 2014).

In the repair process following myocardial infarction, FSTL1 promotes the transformation of cardiac fibroblasts into myofibroblasts, enhancing ECM deposition, particularly increasing collagen and fibronectin in the myocardial interstitium. This contributes to improving the mechanical stability of the heart and preventing cardiac rupture (Maruyama et al., 2016). However, in the later stages of certain diseases, FSTL1-induced fibrosis may shift into a pathological process, limiting tissue and organ function and leading to irreversible pathological changes. While FSTL1 plays a protective role during the acute repair phase, prolonged overstimulation leads to pathological fibrosis by continuously increasing collagen synthesis. Specifically, excessive endurance exercise over a long period may cause cardiovascular damage and myocardial fibrosis (O'Keefe et al., 2012; Patil et al., 2012). His process may be associated with elevated FSTL1 levels and activation of the TGF-β pathway, which further promotes fibrosis and triggers pathological changes (Jin et al., 2018; Nikooie and Samaneh, 2016).

### 3.7 Anti-remodeling role of FSTL1 in cardiac metabolic regulation

The role of FSTL1 in cardiac pathophysiological processes involves metabolic regulation and ventricular remodeling. Studies have shown that myocardial hibernation occurs not only in ischemic cardiomyopathy but also in nonischemic dilated cardiomyopathy. This phenomenon is characterized by compensatory structural and functional changes in the cardiomyocytes, including glycogen accumulation, decreased capillary density, and metabolic adaptation (Lionetti et al., 2014). The structural and molecular characteristics of myocardial hibernation can be observed even in patients with ischemic cardiomyopathy and dilated cardiomyopathy with patent coronary arteries. This suggests that myocardial remodeling is not solely related to coronary ischemia but may also be driven by microvascular ischemia and metabolic disturbances. Such adaptive changes contribute to the survival of the myocardium under prolonged stress but may also lead to functional deterioration. Further studies have shown that chronic FSTL1 infusion can stabilize cardiac free fatty acid, glucose, and ketone body metabolism, thereby improving the overall respiratory quotient and systolic and diastolic functions of the heart (Seki et al., 2018). This indicates that FSTL1 exerts an anti-remodelling effect by modulating metabolic adaptability, thereby promoting the recovery of cardiac function. Specifically, FSTL1 activates fatty acid oxidation and inhibits excessive glucose metabolism by regulating the AMPK signaling pathway, thereby enhancing the flexibility of myocardial energy utilization (Seki et al., 2018; Salt and Hardie, 2017). In addition, FSTL1 prevents the downregulation of medium-chain acyl-CoA dehydrogenase, further indicating its protective role in fatty acid metabolism. In summary, regardless of the presence of coronary artery narrowing, the role of FSTL1 in metabolic and structural regulation is evident in pathological cardiac processes. Its regulatory capacity for cardiac metabolism and anti-remodeling properties suggest its potential for treating both ischemic and nonischemic heart diseases.

## 4 Unresolved controversies in FSTL1 research and possible reasons for conflicting findings

### 4.1 Variability in DIP2A expression across different tissues

DIP2A, one of the key receptors for FSTL1, shows clear differences in expression across tissues, cell types, and disease states. This variability is widely seen as one major reason why FSTL1 studies sometimes reach different—or even opposite—conclusions. Since receptor availability and abundance can shape both signal strength and downstream pathway “bias,” an inconsistent DIP2A expression landscape across tissues could make FSTL1 look functionally different in different disease models.

In the heart, some studies suggest that increased DIP2A expression is linked to the cardioprotective effects of FSTL1. For example, in ischemia–reperfusion injury models, upregulation of DIP2A appears to strengthen FSTL1-mediated protection by promoting angiogenesis and reducing cardiomyocyte apoptosis, which ultimately supports functional recovery (Xi et al., 2021; Ouchi et al., 2010). In this context, higher DIP2A levels are often considered one of the key prerequisites for FSTL1 to exert its repair-promoting effects. By contrast, in cancer-related studies, the relationship between DIP2A expression and function is more complicated. Some reports in gastric cancer, liver cancer, and other tumors have found that upregulation of the DIP2A–FSTL1 signaling axis correlates with poor prognosis or greater tumor aggressiveness, implying that this axis may enhance tumor cell proliferation, migration, and invasion, thereby accelerating disease progression (Kudo-Saito et al., 2024). These findings suggest that even when DIP2A is “high,” the DIP2A–FSTL1 axis can drive very different biological outputs depending on the tissue microenvironment. In endothelial cells, DIP2A is relatively broadly expressed and is mainly tied to angiogenesis and vascular repair. Some evidence indicates that the presence of DIP2A helps FSTL1 promote endothelial proliferation and migration through pathways such as Smad signaling (Xi et al., 2021). Importantly, in certain tissues or pathological conditions, insufficient DIP2A expression—or disease-related changes in its regulation—may weaken FSTL1 signaling or redirect it toward alternative downstream effects. This adds another layer of difficulty when comparing results across models or trying to pin down a single mechanism.

Overall, the tissue-specific and pathology-dependent nature of DIP2A expression means that the question of whether “FSTL1 effects are consistent” cannot really be discussed without considering the tissue context and receptor availability. Going forward, a more systematic mapping of DIP2A expression patterns (and the factors that regulate them) across tissues and cell types would help reconcile conflicting findings in the literature and improve the predictability and translational potential of targeting FSTL1 therapeutically.

### 4.2 Lack of consensus on activin-binding sites

Activin is an important cytokine in the TGF-β superfamily, and one classic way it’s regulated is by being neutralized after binding to specific binding proteins like follistatin, which then dampens downstream signaling. But when it comes to whether FSTL1 can directly bind activin—especially Activin A—and regulate it in a functional way, there still isn’t a clear consensus.

Follistatin has been firmly shown to bind activin and block its signaling. However, even though FSTL1 contains a follistatin-like domain, there’s still no definitive evidence that it can bind Activin A the way FST does. Some studies lean toward the idea that FSTL1 is more likely acting indirectly—through other receptors or binding partners—and shaping the broader TGF-β/BMP signaling network, rather than functioning as a classic “activin-neutralizing” protein (Geng et al., 2011). On the other hand, structural and functional reviews also point out that FSTL1 is involved in regulating multiple related pathways, but its exact binding interface, interaction partners, and the full mechanistic chain still have not been mapped out in a comprehensive way (Parfenova et al., 2021). Because of that, the controversy around the “FSTL1–activin” relationship does not just affect how we think FSTL1 modulates TGF-β family signaling—it can also lead different studies to attribute the mechanism to different pathways. Going forward, more detailed structural biology work and stronger functional validation will be needed to pin down whether direct binding truly exists and, if it does, what it actually means biologically.

### 4.3 Conflicting findings in fibrosis models

In cardiac fibrosis research, FSTL1 shows a pretty clear “two-way phenotype”—meaning that under different experimental conditions, it can be linked to either pro-fibrotic outcomes or anti-fibrotic outcomes. This has become one of the major controversies in the field. A big part of the problem is that fibrosis itself is a double-edged sword: it’s essential for repair (scar formation and preventing rupture), but if it stays switched on for too long, it can turn into maladaptive remodeling. FSTL1 may also shift its dominant effects depending on the stage of disease and the specific pathological context.

On one side, evidence for a pro-fibrotic, pro-repair role mainly comes from post–myocardial infarction healing settings. FSTL1 can be strongly induced in fibroblasts and other matrix-related cells, and it promotes fibroblast activation and migration—effects that are generally associated with collagen deposition and stabilizing the structure of the injured region. When FSTL1 is lost, fibrosis-related genes in the damaged area (such as Collagen I and Fibronectin) drop, and fibroblast migratory capacity is reduced, suggesting that FSTL1 helps drive “reparative” fibrosis during healing (Maruyama et al., 2016). On the other side, some studies report that FSTL1 can be anti-fibrotic and protective in certain contexts. For instance, in ischemic cardiac injury models complicated by diabetes, exogenous supplementation or overexpression of FSTL1 has been shown to reduce fibrosis markers and improve cardiac function through the USP10/Notch1 axis, implying that in some metabolically abnormal settings, FSTL1 may tilt toward a more “anti-fibrotic/protective” output (Lu et al., 2021). Taken together, these differences suggest that FSTL1’s role in fibrosis isn’t as simple as “pro” versus “anti.” Instead, it likely depends on a mix of factors—such as the type of model (infarction vs pressure overload), the time window being studied (acute repair vs chronic remodeling), and whether there are additional layers like metabolic dysfunction or inflammatory status. Going forward, studies that more deliberately stratify by time, tissue context, and disease background will be essential for pinning down when and why FSTL1 flips between these two directions, and what the key molecular switches actually are.

### 4.4 Glycosylation-dependent functional divergence

FSTL1 is a secreted glycoprotein, and its glycosylation status can influence how stable the protein’s structure is, how it interacts with receptors or binding partners, and which downstream pathways it tends to favor. That makes it a very plausible explanation for why “it’s still FSTL1, but the biological outcome isn’t the same.” This issue shows up especially clearly in studies on cardiac regeneration and injury repair: depending on the experimental system, FSTL1 can look more pro-proliferative, more broadly cytoprotective, or even seem to produce opposite conclusions—and differences in glycosylation are often seen as one of the key variables behind that (Magadum et al., 2018; Mattiotti et al., 2018).

Some work suggests that N-linked glycosylation at specific sites on FSTL1 (for example, N180) can be a deciding factor for its ability to promote cardiomyocyte proliferation and repair. Using site-directed mutations such as N180Q, researchers have shown that changing the glycosylation state at this site can markedly shift regeneration-related phenotypic outputs (Magadum et al., 2018). Beyond individual sites, differences in expression systems (for instance, bacterial versus mammalian expression) can also create major glycosylation differences, which may help explain why different labs sometimes see different effects. Additional evidence comes from direct comparisons between low-glycosylated (“glylow”) and high-glycosylated (“glyhigh”) FSTL1. In hypoxia-related models, both forms can improve cell survival or injury-related readouts, but the low-glycosylated form tends to trigger stronger proliferation-associated effects. This suggests glycosylation may partially separate two functional “modes” of FSTL1—general cytoprotection versus pro-proliferation/pro-regeneration activity (Peters et al., 2022).

So overall, glycosylation differences are very likely one of the main reasons FSTL1 studies do not always line up. Since FSTL1 glycosylation patterns can shift depending on the producing cell type and its stress state, the downstream signaling output may also tilt between “regeneration/proliferation” and “cell protection plus ECM remodeling,” which can lead to different—or even opposite—phenotypes across models. Going forward, explicitly factoring glycosylation into cross-study comparisons should make it easier to interpret the conflicting results in a more coherent way.

### 4.5 Context-dependent effects

FSTL1’s role after myocardial injury is highly context-dependent. Metabolic background and ischemic burden can work together to shape inflammation levels, cellular stress responses, and the overall repair program. As a result, they can shift FSTL1’s net effect between “protection/repair” and “maladaptive remodeling,” which helps explain why different studies sometimes reach opposite conclusions.

In the setting of diabetes or metabolic syndrome, factors like insulin resistance, chronic low-grade inflammation, and oxidative stress may change baseline FSTL1 levels and even the direction of its downstream effects. Clinical and population-based studies have linked circulating FSTL1 to metabolic syndrome and insulin resistance, suggesting that it is regulated by metabolic status (Yang et al., 2021). In addition, in specific models of “MI plus type 2 diabetes,” exogenous supplementation or overexpression of FSTL1 reduced myocardial fibrosis and improved cardiac function through the USP10/Notch1 axis. This supports the idea that under certain metabolic-abnormal conditions, FSTL1 may lean more toward an “anti-fibrotic/protective” output (Lu et al., 2021). On the other hand, the severity of ischemia and the time window being studied can also define where the “benefit vs harm” boundary falls for FSTL1 signaling. Multiple studies show that FSTL1 is induced after ischemia–reperfusion or myocardial infarction, and giving exogenous FSTL1 in animal I/R models can reduce infarct size and lessen injury—suggesting it may be protective early in the acute damage phase (Ogura et al., 2012). However, clinical studies have also reported that in some MI patients, myocardial-derived FSTL1 remains elevated for a longer period, and this persistent elevation is associated with adverse left ventricular remodeling and worsening function. That implies that when injury burden is higher or when repair shifts into a chronic remodeling stage, FSTL1 may be more reflective of (or potentially involved in) maladaptive remodeling (Uematsu et al., 2020). So overall, differences in metabolic environment, along with differences in ischemic load and observation time window, likely act together to steer the downstream effects of FSTL1. These context factors are a key part of why the same molecule can look protective in one study and harmful in another.

## 5 Potential therapeutic applications of FSTL1

FSTL1 has drawn considerable attention as a therapeutic candidate due to its pleiotropic actions in cardioprotection, inflammation control, and vascular remodeling. Several preclinical approaches have been explored. Epicardial delivery of recombinant FSTL1 protein or gene vectors has been shown to enhance cardiomyocyte survival and angiogenesis after MI, whereas loss-of-function models exacerbate myocardial injury (Wei et al., 2015). More recently, advanced strategies have been developed to improve delivery and disease specificity. In a diabetic MI model, AAV9-mediated cardiac-specific FSTL1 expression significantly reduced apoptosis and fibrosis, underscoring both the therapeutic potential and the need for context-dependent modulation (Lu et al., 2021). Likewise, systematic reviews have summarized that FSTL1-driven angiogenesis engages multiple downstream pathways (see Section ‘Pro-angiogenic role of FSTL1’ and Figure 3), providing diverse targets for intervention (Karisa et al., 2025). Beyond gene and protein supplementation, lifestyle interventions may serve as non-pharmacological modulators of FSTL1. Acute high-intensity interval exercise markedly elevates circulating FSTL1 levels, suggesting that tailored exercise prescriptions could complement pharmacological strategies (Ji et al., 2024).

Despite these opportunities, key translational barriers and safety considerations must be addressed before FSTL1 can be advanced as a post-MI therapy. First, the dose–response relationship remains insufficiently characterized, and it is unclear whether therapeutic benefit follows a monotonic curve or a narrow effective window. Second, FSTL1 appears to exhibit time-dependent effects: augmentation during the acute/subacute phase may favor cardiomyocyte protection and reparative angiogenesis, whereas sustained activation during the remodeling phase could shift signaling toward fibroblast activation, extracellular matrix accumulation, and maladaptive fibrosis. Third, these temporal dynamics raise an important safety concern—long-term or excessive FSTL1 overexpression may increase fibrotic risk—highlighting the need for strategies that enable titratable and transient exposure. Fourth, delivery specificity remains a central challenge. While AAV9-based cardiac gene transfer and epicardial biomaterial patches have shown promise, further development of cardiac-targeted nanoparticles and localized epicardial platforms will be required to minimize systemic exposure and improve myocardial selectivity. Fifth, given FSTL1’s pro-angiogenic activity, systemic leakage may carry a risk of off-target angiogenesis in non-cardiac vascular beds; therefore, biodistribution profiling and vascular safety endpoints should be incorporated into preclinical development. Sixth, how FSTL1-based interventions interact with guideline-directed post-MI pharmacotherapy (e.g., β-blockers and ACE inhibitors/ARBs) remains unknown; dedicated combination studies are needed to define whether standard therapies modify FSTL1 signaling outputs or alter its therapeutic window.

Importantly, two emerging directions may help overcome these limitations. One is glycoform-informed FSTL1 biologics. Evidence that epicardial-derived FSTL1 displays regenerative activity that is not consistently recapitulated by myocardial-derived FSTL1 supports the concept that post-translational modifications—particularly N-glycosylation—may determine pro-regenerative versus pro-fibrotic bias. In line with this, recombinant or engineered FSTL1 variants with defined glycosylation status (e.g., ablation of the N180 glycosylation site; N180Q) have been reported to promote cardiomyocyte cell-cycle entry and improve post-MI repair, suggesting that “glycoform-defined” FSTL1 may provide a more favorable benefit–risk profile than indiscriminate overexpression (Wei et al., 2015; Magadum et al., 2018). The second direction is RNA-based therapy, particularly modified mRNA (modRNA). Compared with viral vectors, modRNA enables rapid, transient, and titratable protein expression, which may better match the time-sensitive nature of post-MI repair and mitigate concerns related to long-term overexpression–associated fibrosis. Engineered FSTL1 modRNA approaches (including glycosylation-informed variants) therefore represent a compelling, clinically actionable strategy for short-course treatment during the early reparative window.

## 6 Conclusion and outlook

Collectively, the available evidence positions FSTL1 as a multi-node regulator of post-MI healing rather than a single-pathway effector. Across experimental systems, FSTL1 enhances cardiomyocyte survival and stress tolerance (notably via AMPK- and PI3K/Akt-associated signaling), shapes the inflammatory milieu, supports vascular repair, and modulates fibroblast activation and extracellular matrix (ECM) remodeling—thereby influencing both scar stabilization and functional recovery. Mechanistically, these actions map onto interconnected signaling networks (BMP/SMAD, PI3K/Akt/mTOR, MAPK/ERK, and TGF-β/Smad2/3), consistent with the concept that FSTL1 acts as an upstream “coordination signal” coupling immune resolution, angiogenesis, and remodeling.

Despite this promise, clinical translation remains limited by several unresolved controversies that require explicit resolution. The most prominent debate is whether FSTL1 is predominantly reparative or pro-fibrotic. Current data support a context- and time-dependent switch: FSTL1 may facilitate the reparative fibrosis required to prevent rupture, whereas sustained signaling could promote maladaptive fibroblast activation and excessive ECM accumulation. A second issue concerns molecular “identity.” Differences in glycosylation—including N-glycosylation at sites such as N180—can shift functional bias toward regeneration/proliferation versus cytoprotection and ECM remodeling, offering a plausible explanation for divergent outcomes across models. Third, uncertainty persists at the receptor level, including tissue- and disease-dependent availability of functional receptors (e.g., DIP2A) and incomplete clarity regarding proposed binding partners such as activin.

These controversies define the key knowledge gaps moving forward: (i) delineating the dominant cellular sources, receptor usage, and post-translationally defined FSTL1 species across repair phases; (ii) establishing dose–response relationships and therapeutic time windows that maximize benefit while minimizing late fibrosis; (iii) characterizing biodistribution, potential off-target angiogenic effects, and long-term safety; and (iv) clarifying how FSTL1-based interventions interact with guideline-directed post-MI pharmacotherapy and common comorbidities.

Looking ahead, we propose that the field should shift from “more FSTL1” to “precision FSTL1.” Priorities include time-resolved, cell-type–specific profiling (single-cell and spatial multi-omics), glycoform-informed biologics (engineered recombinant FSTL1 variants), and transient, titratable delivery platforms (e.g., modRNA) aligned with the reparative window to mitigate risks associated with chronic overexpression. In parallel, next-generation cardiac-targeted delivery strategies (optimized AAV9, epicardial patches, and nanoparticles) and rigorous combination studies with standard therapies should be integrated early to accelerate translation. If these precision and translational challenges are addressed, FSTL1 could plausibly progress from a mechanistic marker of remodeling to a clinically actionable target—and a biomarker for post-MI risk stratification and repair augmentation.

## References

[B1] Adams D. Larman B. Oxburgh L. (2007). Developmental expression of mouse Follistatin-like 1 (Fstl1): dynamic regulation during organogenesis of the kidney and lung. Gene Expression Patterns GEP 7 (4), 491–500. 10.1016/j.modgep.2006.10.009 17129766 PMC4043338

[B94] Aikawa T. Shimada K. Miyauchi K. Miyazaki T. Sai E. Ouchi S. (2019). Associations among circulating levels of follistatin-like 1, clinical parameters, and cardiovascular events in patients undergoing elective percutaneous coronary intervention with drug-eluting stents. PLOS ONE 14 (4), e0216297. 10.1371/journal.pone.0216297 31034503 PMC6488088

[B2] Arabzadeh E. Samadian Z. Tofighi A. Tolouei Azar J. (2020). Alteration of follistatin-like 1, neuron-derived neurotrophic factor, and vascular endothelial growth factor in diabetic cardiac muscle after moderate-intensity aerobic exercise with insulin. Sport Sci. Health 16, 491–499.

[B3] Bamberger C. Schärer A. Antsiferova M. Tychsen B. Pankow S. Müller M. (2005). Activin controls skin morphogenesis and wound repair predominantly via stromal cells and in a concentration-dependent manner *via* keratinocytes. Am. Journal Pathology 167 (3), 733–747. 10.1016/S0002-9440(10)62047-0 16127153 PMC1698729

[B4] Braasch W. Gudbjarnason S. Puri P. S. Ravens K. G. Bing R. J. (1968). Early changes in energy metabolism in the myocardium following acute coronary artery occlusion in anesthetized dogs. Circulation Research 23 (3), 429–438. 10.1161/01.res.23.3.429 5676453

[B5] Chaly Y. Hostager B. Smith S. Hirsch R. (2014a). Follistatin-like protein 1 and its role in inflammation and inflammatory diseases. Immunol. Research 59 (1-3), 266–272. 10.1007/s12026-014-8526-z 24838142

[B6] Chaly Y. Fu Y. Marinov A. Hostager B. Yan W. Campfield B. (2014b). Follistatin-like protein 1 enhances NLRP3 inflammasome-mediated IL-1β secretion from monocytes and macrophages. Eur. Journal Immunology 44 (5), 1467–1479. 10.1002/eji.201344063 24470197 PMC4004659

[B7] Chang H. Brown C. W. Matzuk M. M. (2002). Genetic analysis of the mammalian transforming growth factor-beta superfamily. Endocr. Reviews 23 (6), 787–823. 10.1210/er.2002-0003 12466190

[B8] Cunningham F. Achuthan P. Akanni W. Allen J. Amode M. R. Armean I. M. (2019). Ensembl 2019. Nucleic Acids Research 47 (D1), D745–d751. 10.1093/nar/gky1113 30407521 PMC6323964

[B9] Davis J. Myofibroblasts M. J. D. (2014). Trust your heart and let fate decide. J. Molecular Cellular Cardiology 70, 9–18. 10.1016/j.yjmcc.2013.10.019 PMC3995855 24189039

[B10] Du R. Li K. Guo K. Chen Z. Han L. Bian H. (2024). FSTL1: a double-edged sword in cancer development. Gene 906, 148263. 10.1016/j.gene.2024.148263 38346455

[B11] Fraccarollo D. Galuppo P. Bauersachs J. (2012). Novel therapeutic approaches to post-infarction remodelling. Cardiovasc. Research 94 (2), 293–303. 10.1093/cvr/cvs109 22387461

[B12] Frangogiannis N. G. (2015). Pathophysiology of myocardial infarction. Compr. Physiol. 5 (4), 1841–1875. 10.1002/cphy.c150006 26426469

[B13] Geng Y. Dong Y. Yu M. Zhang L. Yan X. Sun J. (2011). Follistatin-like 1 (Fstl1) is a bone morphogenetic protein (BMP) 4 signaling antagonist in controlling mouse lung development. Proc. Natl. Acad. Sci. United States of America 108 (17), 7058–7063. 10.1073/pnas.1007293108 21482757 PMC3084141

[B14] Hambrock H. O. Kaufmann B. Müller S. Hanisch F. G. Nose K. Paulsson M. (2004). Structural characterization of TSC-36/Flik: analysis of two charge isoforms. J. Biological Chemistry 279 (12), 11727–11735. 10.1074/jbc.M309318200 14701841

[B15] Hayakawa S. Ohashi K. Shibata R. Kataoka Y. Miyabe M. Enomoto T. (2015). Cardiac myocyte-derived follistatin-like 1 prevents renal injury in a subtotal nephrectomy model. J. Am. Soc. Nephrol. JASN 26 (3), 636–646. 10.1681/ASN.2014020210 25071081 PMC4341480

[B16] Hikoso S. Yamaguchi O. Nakano Y. Takeda T. Omiya S. Mizote I. (2009). The IκB kinase β/nuclear factor κB signaling pathway protects the heart from hemodynamic stress mediated by the regulation of manganese superoxide dismutase expression. Circulation Research 105 (1), 70–79. 10.1161/CIRCRESAHA.108.193318 19478205

[B17] Horak M. Fairweather D. Kokkonen P. Bednar D. Bienertova-Vasku J. (2022). Follistatin-like 1 and its paralogs in heart development and cardiovascular disease. Heart Failure Reviews 27 (6), 2251–2265. 10.1007/s10741-022-10262-6 35867287 PMC11140762

[B18] Hu S. Liu H. Hu Z. Li L. Yang Y. (2020). Follistatin-like 1: a dual regulator that promotes cardiomyocyte proliferation and fibrosis. J. Cellular Physiology 235 (9), 5893–5902. 10.1002/jcp.29588 32017077

[B19] Ji M. Cho C. Lee S. (2024). Acute effect of exercise intensity on circulating FGF-21, FSTL-1, cathepsin B, and BDNF in young men. J. Exercise Science Fitness 22 (1), 51–58. 10.1016/j.jesf.2023.11.002 38074189 PMC10698539

[B20] Jiang W. Xiong Y. Li X. Yang Y. (2021). Cardiac fibrosis: cellular effectors, molecular pathways, and exosomal roles. Front. Cardiovascular Medicine 8, 715258. 10.3389/fcvm.2021.715258 34485413 PMC8415273

[B21] Jin Y. K. Li X. H. Wang W. Liu J. Zhang W. Fang Y. S. (2018). Follistatin-like 1 promotes bleomycin-induced pulmonary fibrosis through the transforming growth factor beta 1/Mitogen-Activated protein kinase signaling pathway. Chin. Medical Journal 131 (16), 1917–1925. 10.4103/0366-6999.238151 30082522 PMC6085847

[B22] Jung M. Dodsworth M. Thum T. (2018). Inflammatory cells and their non-coding RNAs as targets for treating myocardial infarction. Basic Res. Cardiol. 114 (1), 4. 10.1007/s00395-018-0712-z 30523422 PMC6290728

[B23] Karisa P. Sylviana N. Fitria N. Setiawan S. (2025). FSTL-1 as a novel cardiokine of cardiac angiogenesis: a systematic review. Vasc. Health Risk Management 21, 437–449. 10.2147/VHRM.S509482 40453420 PMC12126109

[B24] Kawabata D. Tanaka M. Fujii T. Umehara H. Fujita Y. Yoshifuji H. (2004). Ameliorative effects of follistatin-related protein/TSC-36/FSTL1 on joint inflammation in a mouse model of arthritis. Arthritis Rheumatism 50 (2), 660–668. 10.1002/art.20023 14872511

[B25] Kerkelä R. (2022). Hypo-glycosylated follistatin-like 1 for new cardiomyocyte formation. Mol. Ther. Methods Clin. Dev. 25, 331–332. 10.1016/j.omtm.2022.04.005 35615706 PMC9114625

[B26] Kretzschmar K. Post Y. Bannier-Hélaouët M. Mattiotti A. Drost J. Basak O. (2018). Profiling proliferative cells and their progeny in damaged murine hearts. Proc. Natl. Acad. Sci. United States of America 115 (52), E12245–e12254. 10.1073/pnas.1805829115 30530645 PMC6310797

[B27] Kübler W. Spieckermann P. G. (1970). Regulation of glycolysis in the ischemic and the anoxic myocardium. J. Molecular Cellular Cardiology 1 (4), 351–377. 10.1016/0022-2828(70)90034-9 4937794

[B28] Kudo-Saito C. Matsumura S. Mori T. Honma Y. Yoshimoto S. J. A. J. o.C. R. (2024). Prognostic significance of the FSTL1-DIP2A axis in early-stage tongue. Cancer 14 (8), 3816–3825. 10.62347/RZAO3562 39267678 PMC11387867

[B29] Lara-Pezzi E. Felkin L. E. Birks E. J. Sarathchandra P. Panse K. D. George R. (2008). Expression of follistatin-related genes is altered in heart failure. Endocrinology 149 (11), 5822–5827. 10.1210/en.2008-0151 18617621

[B30] Li L. Li X. Liu X. Dong Y. Geng Y. Liu X. (2013). Characterization, and preliminary X-ray crystallographic analysis of recombinant murine Follistatin-like 1 expressed in drosophila S2 cells. Biosci. Trends 7 (2), 93–100. 23612079

[B31] Lionetti V. Matteucci M. Ribezzo M. Di Silvestre D. Brambilla F. Agostini S. (2014). Regional mapping of myocardial hibernation phenotype in idiopathic end-stage dilated cardiomyopathy. J. Cellular Molecular Medicine 18 (3), 396–414. 10.1111/jcmm.12198 24444256 PMC3955147

[B32] Liu S. Shen H. Xu M. Liu O. Zhao L. Liu S. (2010). FRP inhibits ox-LDL-induced endothelial cell apoptosis through an Akt-NF-{kappa}B-Bcl-2 pathway and inhibits endothelial cell apoptosis in an apoE-knockout mouse model. Am. Journal Physiology. Endocrinol. Metabolism 299 (3), E351–E363. 10.1152/ajpendo.00005.2010 20530739

[B33] Liu X. Wang L. Wang Y. Qiao X. Chen N. Liu F. (2024). Myocardial infarction complexity: a multi-omics approach. Clin. Chim. Acta; Int. J. Clin. Chem. 552, 117680. 10.1016/j.cca.2023.117680 38008153

[B34] Liu Y. Sun X. Jia Z. Hou Q. Yuan M. Xu T. (2025). P2Y6 promoted pruning of FSTL1 nerves by cutaneous macrophages to reset pain threshold and cardiac function. Purinergic Signalling 21, 621–635. 10.1007/s11302-025-10088-5 40293604 PMC12454787

[B35] Lu L. Ma J. Liu Y. Shao Y. Xiong X. Duan W. (2021). FSTL1-USP10-Notch1 signaling axis protects against cardiac dysfunction through inhibition of myocardial fibrosis in diabetic mice. Front. Cell Dev. Biol. 9, 757068. 10.3389/fcell.2021.757068 34957094 PMC8695978

[B36] Magadum A. Singh N. Kurian A. A. Sharkar M. T. K. Chepurko E. Zangi L. J. M. T. N. A. Ablation of a single N-glycosylation site in human FSTL 1 induces cardiomyocyte proliferation and cardiac regeneration, Mol. Ther. Nucleic Acids 13:133–143. (2018). 10.1016/j.omtn.2018.08.021 30290305 PMC6171324

[B37] Martin J. L. Demiralp B. Bolinger K. J. J. o.C. F. (2022). The persistent heart failure and mortality burden following anterior STEMI treated with PCI; the role of SSO2 therapy. J. Card. Fail. 28 (5), S133. 10.1016/j.cardfail.2022.03.343

[B38] Maruyama S. Nakamura K. Papanicolaou K. N. Sano S. Shimizu I. Asaumi Y. (2016). Follistatin-like 1 promotes cardiac fibroblast activation and protects the heart from rupture. EMBO Molecular Medicine 8 (8), 949–966. 10.15252/emmm.201506151 27234440 PMC4967946

[B39] Mattiotti A. Prakash S. Barnett P. van den Hoff M. J. J. C. Sciences M. L. (2018). Follistatin-like 1 in development and human diseases. Cell. Mol. Life Sci. 75 (13), 2339–2354. 10.1007/s00018-018-2805-0 29594389 PMC5986856

[B40] Mayer K. Buchbinder A. Morty R. E. (2012). Activin A: a mediator governing inflammation, immunity, and repair. Am. J. Respir. Crit. Care Med. 185 (4), 350–352. 10.1164/rccm.201112-2210ED 22336674

[B41] Miyabe M. Ohashi K. Shibata R. Uemura Y. Ogura Y. Yuasa D. (2014). Muscle-derived follistatin-like 1 functions to reduce neointimal formation after vascular injury. Cardiovasc. Research 103 (1), 111–120. 10.1093/cvr/cvu105 24743592 PMC4834864

[B42] Miyamae T. Marinov A. D. Sowders D. Wilson D. C. Devlin J. Boudreau R. (2006). Follistatin-like protein-1 is a novel proinflammatory molecule. J. Immunol. 177 (7), 4758–4762. 10.4049/jimmunol.177.7.4758 16982916

[B43] Moran A. E. Forouzanfar M. H. Roth G. A. Mensah G. A. Ezzati M. Flaxman A. (2014). The global burden of ischemic heart disease in 1990 and 2010: the global burden of disease 2010 study. Circulation 129 (14), 1493–1501. 10.1161/CIRCULATIONAHA.113.004046 24573351 PMC4181601

[B44] Nikooie R. Samaneh S. (2016). Exercise-induced lactate accumulation regulates intramuscular triglyceride metabolism via transforming growth factor-β1 mediated pathways. Mol. Cellular Endocrinology 419, 244–251. 10.1016/j.mce.2015.10.024 26522131

[B45] O'Keefe J. H. Patil H. R. Lavie C. J. Magalski A. Vogel R. A. McCullough P. A. (2012). Potential adverse cardiovascular effects from excessive endurance exercise. Mayo Clin. Proceedings 87 (6), 587–595. 10.1016/j.mayocp.2012.04.005 PMC3538475 22677079

[B46] Ogura Y. Ouchi N. Ohashi K. Shibata R. Kataoka Y. Kambara T. (2012). Therapeutic impact of follistatin-like 1 on myocardial ischemic injury in preclinical models. Circulation 126 (14), 1728–1738. 10.1161/CIRCULATIONAHA.112.115089 22929303 PMC3548325

[B47] Oshima Y. Ouchi N. Sato K. Izumiya Y. Pimentel D. R. Walsh K. J. C. (2008). Follistatin-like 1 is an Akt-regulated cardioprotective factor that is secreted by the heart. Circulation 117 (24), 3099–3108. 10.1161/CIRCULATIONAHA.108.767673 18519848 PMC2679251

[B48] Ouchi N. Oshima Y. Ohashi K. Higuchi A. Ikegami C. Izumiya Y. (2008). Follistatin-like 1, a secreted muscle protein, promotes endothelial cell function and revascularization in ischemic tissue through a nitric-oxide synthase-dependent mechanism. J. Biol. Chem. 283 (47), 32802–32811. 10.1074/jbc.M803440200 18718903 PMC2583310

[B49] Ouchi N. Asaumi Y. Ohashi K. Higuchi A. Sono-Romanelli S. Oshima Y. (2010). DIP2A functions as a FSTL1 receptor. J. Biol. Chem. 285 (10), 7127–7134. 10.1074/jbc.M109.069468 20054002 PMC2844162

[B50] Parfenova O. K. Kukes V. G. Grishin D. V. (2021). Follistatin-like proteins: structure, functions and biomedical importance. Biomedicines 9 (8), 999. 10.3390/biomedicines9080999 34440203 PMC8391210

[B51] Patil H. R. O'Keefe J. H. Lavie C. J. Magalski A. Vogel R. A. McCullough P. A. (2012). Cardiovascular damage resulting from chronic excessive endurance exercise. Mo. Medicine 109 (4), 312–321. PMC6179786 22953596

[B52] Peters M. C. Di M. S. Boelens T. Qin J. van Mil A. Doevendans P. A. (2022). Follistatin-like 1 promotes proliferation of matured human hypoxic iPSC-cardiomyocytes and is secreted by cardiac fibroblasts, 25, 3–16. 10.1016/j.omtm.2022.02.005 PMC8917270 35317048

[B53] Pfeffer J. M. Pfeffer M. A. Fletcher P. J. Braunwald E. (1991). Progressive ventricular remodeling in rat with myocardial infarction. Am. J. Physiol. 260 (5 Pt 2), H1406–H1414. 10.1152/ajpheart.1991.260.5.H1406 2035662

[B54] Phillips D. J. de Kretser D. M. (1998). Follistatin: a multifunctional regulatory protein. Front. Neuroendocrinol. 19 (4), 287–322. 10.1006/frne.1998.0169 9799587

[B55] Phillips D. J. Jones K. L. Clarke I. J. Scheerlinck J. P. de Kretser D. M. (2005). Activin A: from sometime reproductive factor to genuine cytokine. Vet. Immunol. Immunopathol. 108 (1-2), 23–27. 10.1016/j.vetimm.2005.08.011 16140391

[B56] Pontemezzo E. Foglio E. Vernucci E. Magenta A. D'Agostino M. Sileno S. (2021). miR-200c-3p regulates epitelial-to-mesenchymal transition in epicardial mesothelial cells by targeting epicardial follistatin-related protein 1. Int. J. Mol. Sci. 22 (9), 4971. 10.3390/ijms22094971 34067060 PMC8125323

[B57] Reed G. W. Rossi J. E. Cannon C. P. J. T. L. (2017). Acute myocardial infarction. Acute Myocardial Infarction 389 (10065), 197–210. 10.1016/S0140-6736(16)30677-8 27502078

[B58] Regula K. M. Baetz D. Kirshenbaum L. A. (2004). Nuclear factor-kappaB represses hypoxia-induced mitochondrial defects and cell death of ventricular myocytes. Circulation 110 (25), 3795–3802. 10.1161/01.CIR.0000150537.59754.55 15596562

[B59] Rimphanitchayakit V. Tassanakajon A. (2010). Structure and function of invertebrate Kazal-type serine proteinase inhibitors. Dev. Comparative Immunology 34 (4), 377–386. 10.1016/j.dci.2009.12.004 19995574

[B60] Rosenberg M. I. Georges S. A. Asawachaicharn A. Analau E. Tapscott S. J. (2006). MyoD inhibits Fstl1 and utrn expression by inducing transcription of miR-206. J. Cell Biology 175 (1), 77–85. 10.1083/jcb.200603039 17030984 PMC2064500

[B61] Salt I. P. Hardie D. G. (2017). AMP-activated protein kinase: an ubiquitous signaling pathway with key roles in the cardiovascular system. Circulation Research 120 (11), 1825–1841. 10.1161/CIRCRESAHA.117.309633 28546359 PMC5447810

[B62] Seki M. Powers J. C. Maruyama S. Zuriaga M. A. Wu C. L. Kurishima C. (2018). Acute and chronic increases of circulating FSTL1 normalize energy substrate metabolism in pacing-induced heart failure. Circ. Heart Failure 11 (1), e004486. 10.1161/CIRCHEARTFAILURE.117.004486 29317401 PMC5765881

[B63] Shen H. Cui G. Li Y. Ye W. Sun Y. Zhang Z. (2019). Follistatin-like 1 protects mesenchymal stem cells from hypoxic damage and enhances their therapeutic efficacy in a mouse myocardial infarction model. Stem Cell Research and Therapy 10 (1), 17. 10.1186/s13287-018-1111-y 30635025 PMC6330478

[B64] Shi D. L. Shi G. R. Xie J. Du X. Z. Yang H. (2016). MicroRNA-27a inhibits cell migration and invasion of fibroblast-like synoviocytes by targeting follistatin-like protein 1 in rheumatoid arthritis. Mol. Cells 39 (8), 611–618. 10.14348/molcells.2016.0103 27498552 PMC4990753

[B65] Shibanuma M. Mashimo J. Mita A. Kuroki T. Nose K. (1993). Cloning from a mouse osteoblastic cell line of a set of transforming-growth-factor-beta 1-regulated genes, one of which seems to encode a follistatin-related polypeptide. Eur. Journal Biochemistry 217 (1), 13–19. 10.1111/j.1432-1033.1993.tb18212.x 7901004

[B66] Shimano M. Ouchi N. Nakamura K. van Wijk B. Ohashi K. Asaumi Y. (2011). Cardiac myocyte follistatin-like 1 functions to attenuate hypertrophy following pressure overload. Proc. Natl. Acad. Sci. United States of America 108 (43), E899–E906. 10.1073/pnas.1108559108 21987816 PMC3203781

[B67] Shinde A. V. Frangogiannis N. G. J. J. o.m. cardiology c. (2014). Fibroblasts in myocardial infarction: a role in inflammation and repair, 70, 74–82. 10.1016/j.yjmcc.2013.11.015 PMC3995820 24321195

[B68] Sibinska Z. Tian X. Korfei M. Kojonazarov B. Kolb J. S. Klepetko W. (2017). Amplified canonical transforming growth factor-β signalling *via* heat shock protein 90 in pulmonary fibrosis. Eur. Respiratory Journal 49 (2), 1501941. 10.1183/13993003.01941-2015 28182573

[B69] Squires C. E. Escobar G. P. Payne J. F. Leonardi R. A. Goshorn D. K. Sheats N. J. (2005). Altered fibroblast function following myocardial infarction. J. Molecular Cellular Cardiology 39 (4), 699–707. 10.1016/j.yjmcc.2005.07.008 16111700

[B70] Stelzer G. Rosen N. Plaschkes I. Zimmerman S. Twik M. Fishilevich S. (2016). The GeneCards suite: from gene data mining to disease genome sequence analyses. Curr. Protocols Bioinformatics 54, 1.30.31–31.30.33. 10.1002/cpbi.5 27322403

[B71] Stylianidis V. Hermans K. C. M. Blankesteijn W. M. (2017). Wnt signaling in cardiac remodeling and heart failure. Handb. Experimental Pharmacology 243, 371–393. 10.1007/164_2016_56 27838851

[B72] Sundaram G. M. Common J. E. Gopal F. E. Srikanta S. Lakshman K. Lunny D. P. (2013). See-saw' expression of microRNA-198 and FSTL1 from a single transcript in wound healing. Nature 495 (7439), 103–106. 10.1038/nature11890 23395958

[B73] Sundaram G. M. Ismail H. M. Bashir M. Muhuri M. Vaz C. Nama S. (2017). EGF hijacks miR-198/FSTL1 wound-healing switch and steers a two-pronged pathway toward metastasis. J. Experimental Medicine 214 (10), 2889–2900. 10.1084/jem.20170354 28827448 PMC5626400

[B74] Tanaka M. Murakami K. Ozaki S. Imura Y. Tong X. P. Watanabe T. (2010). DIP2 disco-interacting protein 2 homolog A (drosophila) is a candidate receptor for follistatin-related protein/follistatin-like 1--analysis of their binding with TGF-β superfamily proteins. FEBS Journal 277 (20), 4278–4289. 10.1111/j.1742-4658.2010.07816.x 20860622

[B75] Tanaka K. Valero-Muñoz M. Wilson R. M. Essick E. E. Fowler C. T. Nakamura K. Follistatin-like 1 regulates hypertrophy in heart failure with preserved ejection fraction. 1(4):207–221. (2016). 27430031 10.1016/j.jacbts.2016.04.002 PMC4944656

[B76] Tsao C. W. Aday A. W. Almarzooq Z. I. Anderson C. A. M. Arora P. Avery C. L. (2023). Heart disease and stroke Statistics-2023 update: a report from the American heart association. Circulation 147 (8), e93–e621. 10.1161/cir.0000000000001123 36695182 PMC12135016

[B77] Uematsu M. Nakamura K. Nakamura T. Watanabe Y. Yoshizaki T. Deyama J. (2020). Persistent myocardial production of follistatin-like 1 is associated with left ventricular adverse remodeling in patients with myocardial infarction: myocardial production of FSTL1 in AMI patients. J. Cardiac Failure 26 (8), 733–738. 10.1016/j.cardfail.2020.05.015 32470377

[B78] van den Berg G. Somi S. Buffing A. A. Moorman A. F. van den Hoff M. J. (2007). Patterns of expression of the follistatin and Follistatin-like1 genes during chicken heart development: a potential role in valvulogenesis and late heart muscle cell formation. Anat. Rec. Hob. 290 (7), 783–787. 10.1002/ar.20559 17549728

[B79] Viola M. de Jager S. C. A. Sluijter J. P. G. (2021). Targeting inflammation after myocardial infarction: a therapeutic opportunity for extracellular vesicles? Int. Journal Molecular Sciences 22 (15), 7831. 10.3390/ijms22157831 34360595 PMC8346058

[B80] Wang Y. Li D. Xu N. Tao W. Zhu R. Sun R. (2011). Follistatin-like protein 1: a serum biochemical marker reflecting the severity of joint damage in patients with osteoarthritis. Arthritis Research and Therapy 13 (6), R193. 10.1186/ar3522 22117761 PMC3334643

[B81] Wei K. Serpooshan V. Hurtado C. Diez-Cuñado M. Zhao M. Maruyama S. (2015). Epicardial FSTL1 reconstitution regenerates the adult mammalian heart. Nature 525 (7570), 479–485. 10.1038/nature15372 26375005 PMC4762253

[B82] Wilson D. C. Marinov A. D. Blair H. C. Bushnell D. S. Thompson S. D. Chaly Y. (2010). Follistatin-like protein 1 is a mesenchyme-derived inflammatory protein and may represent a biomarker for systemic-onset juvenile rheumatoid arthritis. Arthritis Rheumatism 62 (8), 2510–2516. 10.1002/art.27485 20506332 PMC2921021

[B83] Wu X. Reboll M. R. Korf-Klingebiel M. Wollert K. C. (2021). Angiogenesis after acute myocardial infarction. Cardiovasc. Research 117 (5), 1257–1273. 10.1093/cvr/cvaa287 33063086

[B84] Xi Y. Gong D. W. Tian Z. (2016). FSTL1 as a potential mediator of exercise-induced cardioprotection in post-myocardial infarction rats. Sci. Reports 6, 32424. 10.1038/srep32424 27561749 PMC5000295

[B85] Xi Y. Hao M. Tian Z. J. (2019). Resistance exercise increases the regulation of skeletal muscle FSTL1 consequently improving cardiac angiogenesis in rats with myocardial infarctions, 1, 78–87.

[B86] Xi Y. Hao M. Liang Q. Li Y. Gong D. W. Tian Z. (2021). Dynamic resistance exercise increases skeletal muscle-derived FSTL1 inducing cardiac angiogenesis *via* DIP2A-Smad2/3 in rats following myocardial infarction. J. Sport Health Science 10 (5), 594–603. 10.1016/j.jshs.2020.11.010 33246164 PMC8500809

[B87] Xiao Y. Zhang Y. Chen Y. Li J. Zhang Z. Sun Y. (2019). Inhibition of MicroRNA-9-5p protects against cardiac remodeling following myocardial infarction in mice. Hum. Gene Therapy 30 (3), 286–301. 10.1089/hum.2018.059 30101604

[B88] Xu X. Zhang T. Mokou M. Li L. Li P. Song J. (2020). Follistatin-like 1 as a novel adipomyokine related to insulin resistance and physical activity. J. Clin. Endocrinol. Metab. 105 (12), dgaa629. 10.1210/clinem/dgaa629 32894773

[B89] Yang S. Dai H. Hu W. Geng S. Li L. Li X. (2021). Association between circulating follistatin-like-1 and metabolic syndrome in middle-aged and old population: a cross-sectional study. Diabetes/metabolism Research Reviews 37 (2), e3373. 10.1002/dmrr.3373 32592413

[B90] Zhang Z. M. Zhang A. R. Xu M. Lou J. Qiu W. Q. (2017). TLR-4/miRNA-32-5p/FSTL1 signaling regulates mycobacterial survival and inflammatory responses in mycobacterium tuberculosis-infected macrophages. Exp. Cell Research 352 (2), 313–321. 10.1016/j.yexcr.2017.02.025 28215633

[B91] Zheng X. Tian S. Li T. Zhang S. Zhou X. Liu Y. (2025). Host FSTL1 defines the impact of stem cell therapy on liver fibrosis by potentiating the early recruitment of inflammatory macrophages. Signal Transduction Targeted Therapy 10 (1), 81. 10.1038/s41392-025-02162-6 40050288 PMC11885662

[B92] Zhong X. Narasimhan A. Silverman L. M. Young A. R. Shahda S. Liu S. (2022). Sex specificity of pancreatic cancer cachexia phenotypes, mechanisms, and treatment in mice and humans: role of activin. J. Cachexia, Sarcopenia Muscle 13 (4), 2146–2161. 10.1002/jcsm.12998 35510530 PMC9397557

[B93] Zhou J. Liao M. Hatta T. Tanaka M. Xuan X. Fujisaki K. (2006). Identification of a follistatin-related protein from the tick Haemaphysalis longicornis and its effect on tick oviposition. Gene 372, 191–198. 10.1016/j.gene.2005.12.020 16517100

